# Changes in the Equilibrium of Standing on One Leg at Various Life Stages

**DOI:** 10.1155/2012/516283

**Published:** 2012-07-31

**Authors:** Shu Morioka, Takahiko Fukumoto, Makoto Hiyamizu, Atsushi Matsuo, Hideaki Takebayashi, Kenzo Miyamoto

**Affiliations:** ^1^Department of Neurorehabilitation, Graduate School of Health Science, Kio University, 4-2-2 Umaminaka, Koryo, Kitakatsuragigun, Nara 635-0832, Japan; ^2^Department of Physical Therapy, Faculty of Health Science, Kio University, 4-2-2 Umaminaka, Koryo, Kitakatsuragigun, Nara 635-0832, Japan; ^3^Department of Physical Therapy, Tosa Rehabilitation College, 2500-2 Otsu-otsu, Kochi 781-5103, Japan

## Abstract

The ability to maintain a one-leg standing position and the relation between plantar two-point discrimination and standing time on one leg were assessed. Participants were 1,241 apparently healthy people aged 2–92 years. Participants were asked to stand on one leg with eyes open (EO group) or closed (EC group) for up to 120 seconds. Coefficients of determination (COD) between subjects' ages and results for both groups were calculated by quadratic and cubic functions. The slope of the tangent line drawn against the resultant curve was calculated by a differential formula. COD for the quadratic function were 0.65 (EO) and 0.33 (EC); age at maximum values in both groups was 37 years. COD for the cubic function were 0.77 (EO) and 0.52 (EC); maximum values were at ages 30 (EO) and 28 (EC) and minimum values at ages 88 (EO) and 77 (EC). The ability to remain standing on one leg with eyes closed appears to begin deteriorating in the late 20s. Age and plantar two-point discrimination distance had a significant positive correlation, and the two-point discrimination distance and standing time on one leg had a significant negative correlation. Decreased plantar sensation appears to be related to the decline in duration of one-leg standing.

## 1. Introduction

Children will gain upright postural control equivalent to adults' when they are aged 7–10 years [1–4] or 9–12 years [5] according to various studies. The reason may be that children aged over 6 years can appropriately integrate the afferent sensory information required for posture control [6] and acquire the same upright postural control strategy as do adults' [1]. Foudriat et al. [7] revealed that upright postural control in children up to 3 years of age is vision-dominant, but from that age onward, control will be gradually shifted to somatosensory-dominant control. Somatosensory-dominant postural control equivalent to that of adults will be achieved at ages over 6 years, which indicates that the development of standing balance may be nearly completed in the early school years. Morioka [8] has reported that the ability to maintain the one-leg standing position with eyes open will dramatically improve in children within the period from late preschool age to early school age, and the improvement will slow down during late school age. That study indicated that the development of standing balance is nonlinear and that it is accelerated beginning at a certain age.

On the other hand, the involutional process of standing balance has been reported to be opposite to the process of developing postural control strategies that change from vision-dominant control to somatosensory-dominant control [9–12]. Such changes include the increased dependence on vision that occurs in elderly people who have difficulty in maintaining standing balance under the condition that somatosensory information is extremely limited [13–16]. In such an involutional process, the postural balance strategy tends to return from a somatosensory-dominant to a vision-dominant strategy. A linear negative correlation between age and standing time on one leg has been reported [17], which indicates that involution of standing balance progresses with age [18]. Additionally, tactile perception has been reported to greatly contribute to positional balance per se, which is slightly influenced by aging [19, 20]. Based on the above background, we supposed that standing balance might show nonlinear rapid development from preschool age to school age and linear involution from a certain age.

The first objective of this study is to determine whether development and involution of standing balance relevant to fall through life, represented by standing time on one leg, are linear or nonlinear, using functions, and to determine the border age of development and involution by calculation. During the developmental process, the strategy to control standing balance changes from vision-dominant to somatosensory-dominant, while in the involutional process, the strategy changes from somatosensory-dominant to vision-dominant. This suggests the possibility that functional changes of somatic sensation due to aging may affect standing time on one leg. The second objective is to clarify the relation between plantar sensation and standing time on one leg, representing somatic sensations by plantar two-point discrimination.

## 2. Participants

Subjects were 1,241 local residents aged 2 to 92 years who were without orthopedic and nervous disease or history of nervous system disease at the time of measurement and who agreed to participate or whose parents or guardians gave consent for them to participate in this study. In addition, preschool and school age participants were required to be apparently healthy with no past or present serious illnesses or disabilities and to be able to stand with legs together. Measurements of preschool children were done with the permission and cooperation of their kindergarten teachers. Participants were grouped as follows: preschool age (2–6 years), *n* = 167; school age (7–12 years), *n* = 123; adolescence (13–19 years), *n* = 184; 20s, *n* = 196; 30s, *n* = 119; 40s, *n* = 125; 50s, *n* = 95; 60s, *n* = 98; 70s, *n* = 76; 80s, *n* = 42; 90s, *n* = 16.

## 3. Measurement Methods

We used digital stopwatches to measure standing time on one leg. After the measurement with eyes open (hereinafter referred to as “open eyes”), we also measured standing time on one leg with eyes closed (hereinafter referred to as “closed eyes”). The maximum value for the measurement was 120 seconds. As we did not specify which should be weight-bearing leg, the weight-bearing leg was selected by each subject. Each measurement was performed after the measurement staff demonstrated the maneuver. Participants were asked to drop both upper extremities naturally to their sides and to stand on one leg as long as possible. The measurement was completed when any of the following occurred: (1) any change in position of the weight-bearing foot on the floor during the measurement, (2) any body part except the weight-bearing foot touched the floor, and (3) eyes opened during closed eye measurements. Measurements of preschool-age and school-age children as well as middle-aged and elderly people over 50 years were attended by an observer in addition to the measurement staff to prevent subjects from falling.

Subsequent measurements of plantar two-point discrimination were performed in 579 participants ranging from adolescence to 89 years. The two-point discrimination test was performed by measuring the shortest distinguishable distance between 2 points on the sole of the foot. For the measurement, a caliper was placed on the foot with its end at the center of the heel and facing the forefoot.

## 4. Methods of Analysis

We used regression analysis and calculated the Pearson correlation coefficient between age and between measurements with both open and closed eyes. We also determined the coefficients of determination to evaluate fittings of the following equations. Labeling the horizontal axis as age (*x*) and the vertical axis as time (*y*), we estimated linear equations (*y* = *ax* + *b*), quadratic equations (*y* = *ax*2 + *bx* + *c*), and cubic equations (*y* = *ax*3 + *bx*2 + *cx* + *d*). Calculations were performed to determine the points where slopes of tangential lines of the quadratic and cubic curves are zero, based on the theoretical standing time on one leg. In other words, we determined the maximum value of a quadratic curve and maximum and minimum values of a cubic curve. These were calculated by detecting the point where the polarity of the increment of *y* changes, then determining the point where the increment of *y* is zero, based on determined equations. As for precision of calculations, 7 significant figures were used.

Additionally, using mean values of open eyes and closed eyes by age, we calculated the linear equations of mean values (*y*) and ages (*x*) to determine the slopes. We used the Pearson correlation coefficient to evaluate the relation between two-point discrimination and open eyes and closed eyes, respectively. We used two-way ANOVA to compare two-point discrimination data among the age groups from the 10s to 80s.

 In all evaluations, we used a significance level of less than 5%. For calculation of these functions, we used Excel 2003 (Microsoft Co., Ltd.) and Excel Statistics 2002 for Windows (Social Survey Research Information Co., Ltd.), an add-in software. For calculation of maximum values and minimum values, we used Sruler (Fuji Techno Enterprise, Inc.), a graph making software.

## 5. Results

**Figure 1:**
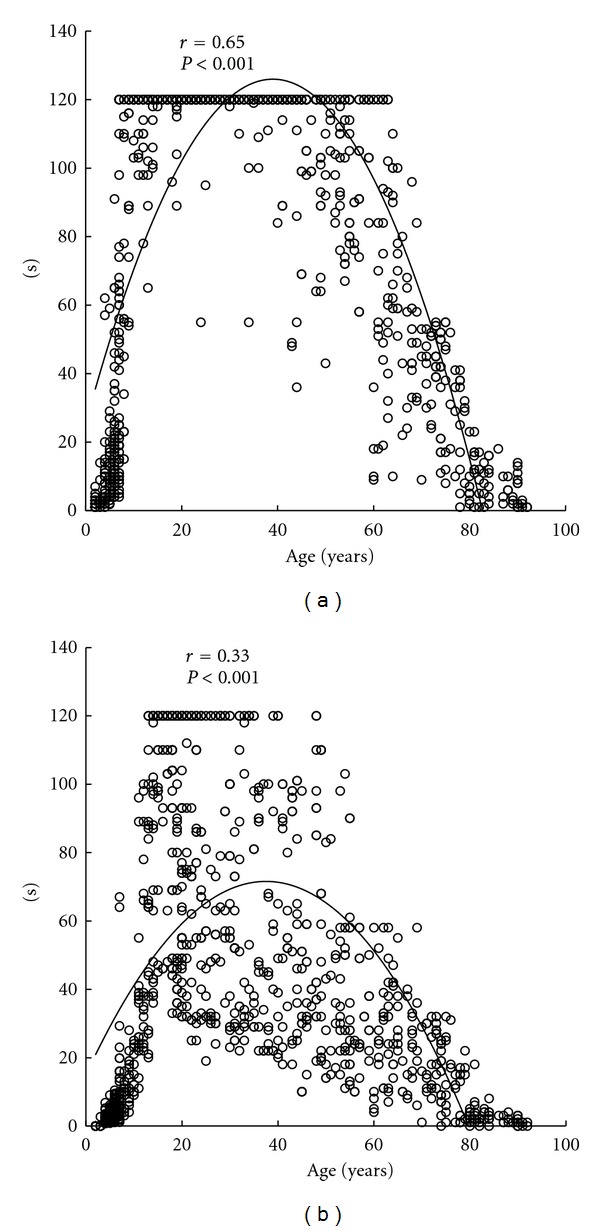
The standing time on one leg versus age for the eyes open (a) and eyes closed (b) along with the respective quadratic regression lines. All data is shown  (*n* = 1241). Ages at the maximum values in quadratic curves were 39.0 years for open eyes and 37.5 years for closed eyes.

**Figure 2:**
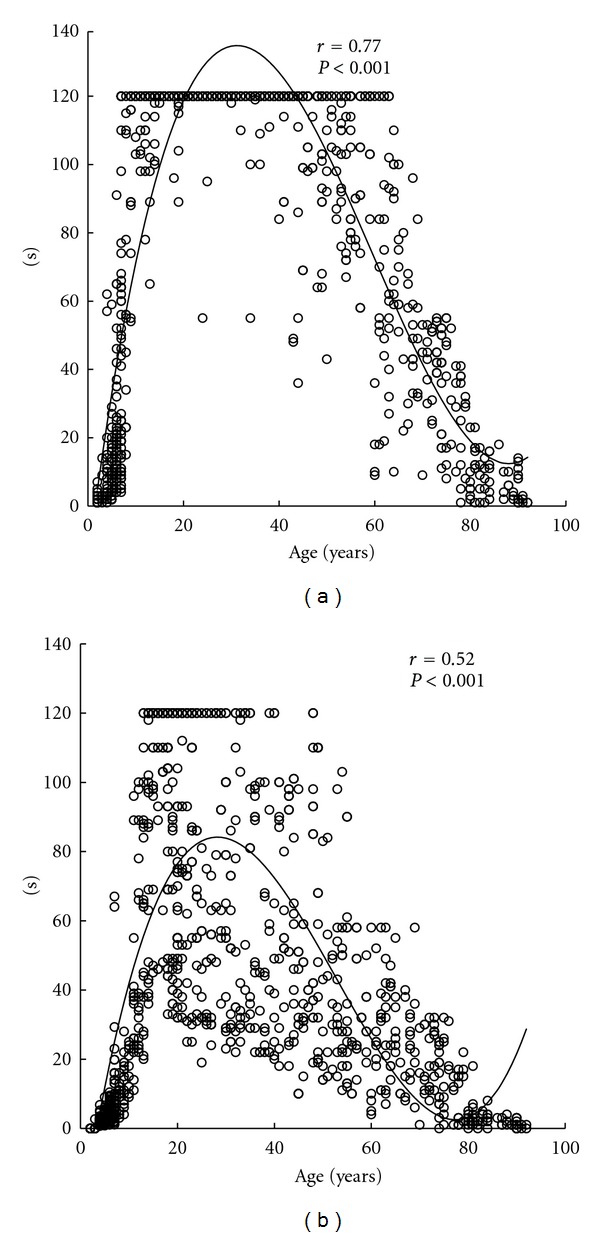
The standing time on one leg versus age for the eyes open (a) and eyes closed (b) along with the respective cubic regression lines. All data is shown (*n* = 1241). Ages at the maximum values in cubic curves were 31.2 years for open eyes and 28.2 years for closed eyes, and ages at the minimum values were 88.1 years for open eyes and 77.3 years for closed eyes.

Coefficients of determination of linear functions for all subjects were 0.02 for open eyes and 0.03 for closed eyes (both, *P* < 0.001). Coefficients of determination of quadratic functions were 0.65 for open eyes and 0.33 for closed eyes (both, *P* < 0.001). Ages at the maximum values in quadratic curves were 39.0 years for open eyes and 37.5 years for closed eyes (Figure 1). Coefficients of determination of cubic functions were 0.77 for open eyes and 0.52 for closed eyes (both, *P* < 0.001). Ages at the maximum values in cubic curves were 31.2 years for open eyes and 28.2 years for closed eyes, and ages at the minimum values were 88.1 years for open eyes and 77.3 years for closed eyes (Figure 2).

**Figure 3:**
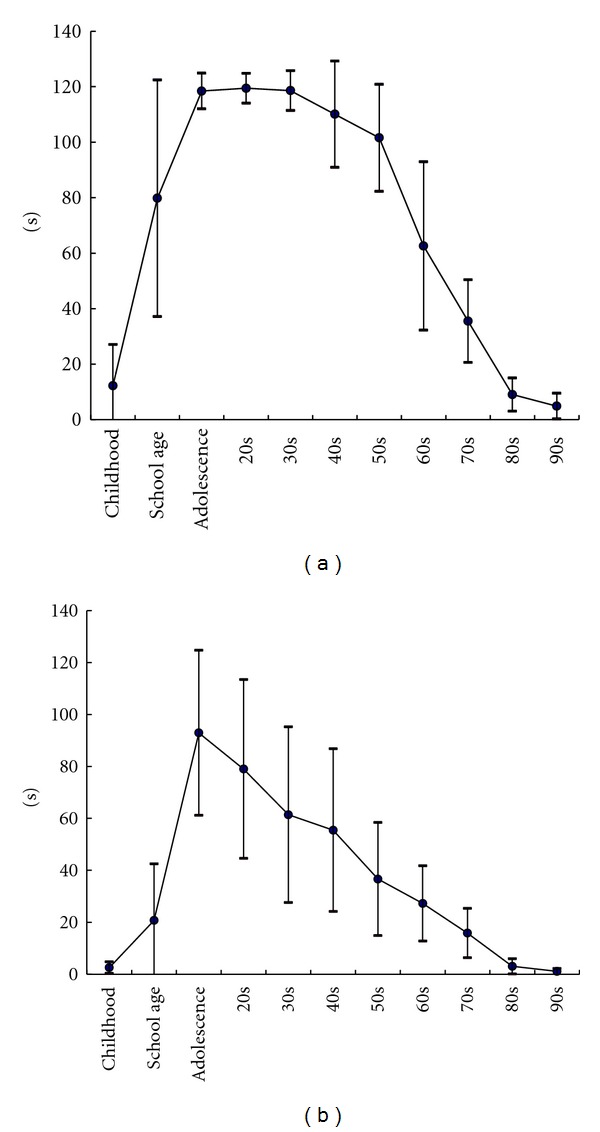
Changes to mean of the standing time on one leg time with the generations. It found the form was similar to trapezoid with its top from adolescence to 30s for open eyes (a). For closed eyes (b), the shape was similar to triangle with its top at adolescence.

Figure 3 shows the means values for standing time for the various age groups. Mean values for standing time with open eyes formed a trapezoidal curve with peak values from adolescence to the 30s (Figure 3(a)). Thus, we divided the linear function for open eyes into 5 stages: (1) from preschool age to adolescence, (2) from adolescence to the 30s, (3) from the 30s to the 50s, (4) from the 50s to the 70s, and (5) from the 70s to the 90s and calculated the slopes with the following results: (1) 7.1; (2) −0.006; (3) −0.86; (4) −3.2; (5) −1.5. For closed eyes, the shape of the curve was triangular with the peak at adolescence (Figure 3(b)). In calculating the slopes, we divided the linear function into 2 stages, (1) from preschool age to adolescence and (2) from adolescence to 90s, with results showing the slope for (1) to be 6.7 and for (2) −1.4. In addition, standard deviations for open eyes were high in those of school age and in their 60s and low from adolescence to the 30s. For closed eyes, standard deviations were relatively high throughout until gradually lessening beginning with those in their 50s.

**Figure 4:**
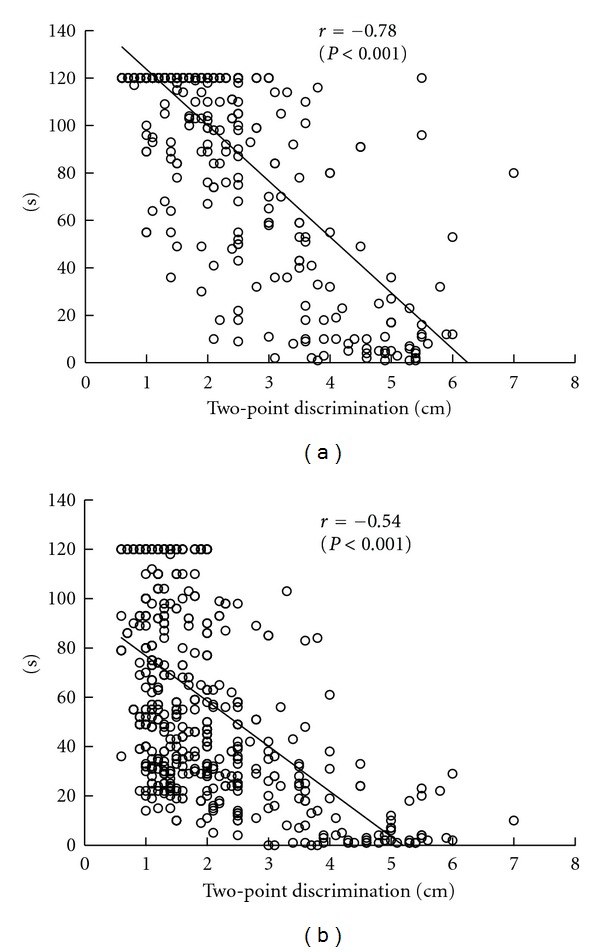
Relation between standing time on one leg and two-point discrimination (*n* = 579). There were significant negative correlations between open eyes (a) and two-point discrimination (*r* = −0.78, *P* < 0.001) and between closed eyes (b) and two-point discrimination (*r* = −0.54, *P* < 0.001).

**Figure 5:**
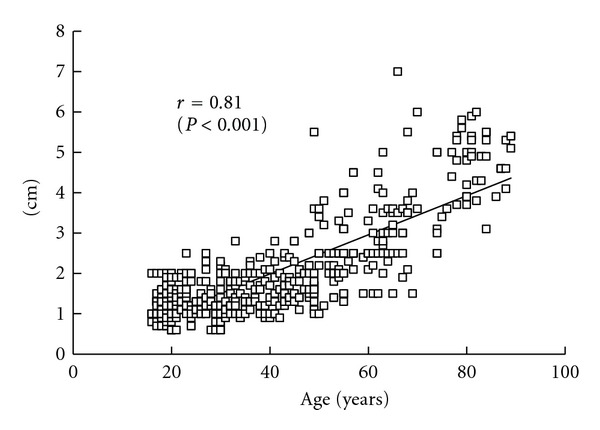
Relation between two-point discrimination and age (*n* = 579). there was a significant positive correlation between age and two-point discrimination (*r* = 0.81, *P* < 0.001).

As shown in Figure 4, there were significant negative correlations between open eyes and two-point discrimination (*r* = −0.78, *P* < 0.001) and between closed eyes and two-point discrimination (*r* = −0.54, *P* < 0.001). Additionally, there was a significant positive correlation between age and two-point discrimination (*r* = 0.81, *P* < 0.001) (Figure 5). A comparison of two-point discrimination distances among the age groups revealed that the distances increased significantly with aging (Table 1).

## 6. Discussion

The first objective of this study was to characterize the process of change in standing time according to age. Coefficient of determination for linear function of age and standing time on one leg was low, indicating that such change was not linear.

 Previous studies have shown that standing time on one leg decreases linearly beginning at a particular age [9, 21–24]. We estimated the age by determining the maximum value. Bohannon et al. [17] reported that the ability to stand on one leg decreases after the age 60. Also, Choy et al. [25] and Pasquier et al. [26] measured posture stability in subjects aged 20 or older and found that stability decreased after the age of 60 years. On the other hand, we found the maximum value for standing time on one leg was shown at 31 years for open eyes and 28 years for closed eyes, which indicates that involution of standing balance begins at these younger ages. This is consistent with results of a previous study that showed that standing balance decreases almost constantly from the 20s to 65s [25, 26]. Anyway, we believe that our study could successfully estimate the time when the involution of standing balance begins by determining the maximum value through calculation. Furthermore, Figure 3 indicates that standing time with closed eyes begins to slowly decline from the 20s whereas the standing time with open eyes starts to drop rapidly from the 60s. These results may be peculiar to conditions of visual blocking in this study.

 The second objective of our study was to characterize the relation between sensory function and standing time. Preceding studies have indicated that causes of involution of standing balance include problems in sensory integration of visual, vestibular, labyrinth, and somatic sensations [27, 28]. It has also been revealed that the decrease in standing time on one leg is more rapid under conditions of closed eyes than open eyes after the age 60 [17]. This indicates that dependence on vision may increase with age [17, 18], and the postural balance strategy may change from somatosensory-dominant into vision-dominant. Based on the fact that somatic sensation around the feet is degraded in elderly people [27, 28], somatic sensation obviously influences standing balance.

We found a significant positive correlation between age and plantar two-point discrimination distance and a significant negative correlation between two-point discrimination distance and standing time on one leg, suggesting that degradation of plantar sensation caused by aging shortened the standing time on one leg. From the interage group comparison which disclosed age-dependent increases in plantar two-point discrimination distances, it was found that aging caused degradation of plantar sensation as well as standing time on one leg and that there was a correlation between plantar sensation and standing time on one leg.

 The wide standard deviations observed for closed eyes in subjects of all ages and for open eyes in subjects in their 60s may be due to wide variations in individual motor function or exercise habits among these subjects. Furthermore, the maximum measurement time of this study was set at 120 seconds, and obtained values, in a sense, are not standard values of balance function itself. In other words, there is a possibility that other factors, including muscle force and muscular endurance, may be involved. In future studies, these issues must be clarified.

However, if we consider standing time on one leg as an inclusive value representing ability of standing balance, our results appear to be valuable in providing standard data on a healthy population based on analysis of extracted results from a considerable number of subjects. Our data can be considered basic data on standing balance function that can be used in health promotion exercises and may be useful in setting a target of standing time on one leg for a patient with impaired standing balance.

## Figures and Tables

**Table 1:** Two-point discrimination distances by age group.

	10s (*n* = 83)	20s (*n* = 154)	30s (*n* = 99)	40s (*n* = 86)	50s (*n* = 55)	60s (*n* = 51)	70s (*n* = 18)	80s (*n* = 33)
Eyes open	1.2 ± 0.3	1.3 ± 0.4	1.4 ± 0.4	1.8 ± 0.7	2.5 ± 0.8	3.0 ± 1.0	4.3 ± 1.1	4.8 ± 0.7

Unit: cm. One-way ANOVA revealed that two-point discrimination distances increased significantly with age (*F* = 217.03, *P* < 0.001).

A multiple comparison test disclosed significant differences between the 10s and 20s, 20s and 30s, and 70s and 80s but not between any other two groups.

## References

[1] Sparto PJ, Redfern MS, Jasko JG, Casselbrant ML, Mandel EM, Furman JM (2006). The influence of dynamic visual cues for postural control in children aged 7-12 years. *Experimental Brain Research*.

[2] Barela JA, Jeka JJ, Clark JE (2003). Postural control in children: coupling to dynamic somatosensory information. *Experimental Brain Research*.

[3] Forssberg H, Nashner LM (1982). Ontogenetic development of postural control in man: adaptation to altered support and visual conditions during stance. *Journal of Neuroscience*.

[4] Shumway-Cook A, Woollacott MH (1985). The growth of stability: postural control from a developmental perspective. *Journal of Motor Behavior*.

[5] Taguchi K, Tada C, Amblard B (1988). Change of body sway with group of children. *Posture and Gait: Development Adaptation and Modulation*.

[6] Shumway-Cook A, Woollacott MH (2001). *Motor Control: Theory and Practical Applications*.

[7] Foudriat BA, Di Fabio RP, Anderson JH (1993). Sensory organization of balance responses in children 3-6 years of age: a normative study with diagnostic implications. *International Journal of Pediatric Otorhinolaryngology*.

[8] Morioka S (2001). Changes in the ability to stand on one leg in children from babyhood to school age. *Rigakuryohogaku*.

[9] Wolfson LI, Whipple R, Amerman P (1985). Gait and balance in the elderly. Two functional capacities that link sensory and motor ability to falls. *Clinics in Geriatric Medicine*.

[10] Horak FB, Shupert CL, Mirka A (1989). Review. Components of postural dyscontrol in the elderly: a review. *Neurobiology of Aging*.

[11] Peterka RJ, Black FO (1990). Age-related changes in human posture control: sensory organization tests. *Journal of Vestibular Research*.

[12] Poulain I, Giraudet G (2008). Age-related changes of visual contribution in posture control. *Gait and Posture*.

[13] Teasdale N, Stelmach GE, Breunig A (1991). Postural sway characteristics of the elderly under normal and altered visual and support surface conditions. *Journals of Gerontology*.

[14] Woollacott MH, Shumway-Cook A, Nashner LM (1986). Aging and posture control: changes in sensory organization and muscular coordination. *International Journal of Aging and Human Development*.

[15] Benjuya N, Melzer I, Kaplanski J (2004). Aging-induced shifts from a reliance on sensory input to muscle cocontraction during balanced standing. *Journals of Gerontology—Series A*.

[16] Lord SR, Menz HB (2000). Visual contributions to postural stability in older adults. *Gerontology*.

[17] Bohannon RW, Larkin PA, Cook AC (1984). Decrease in timed balance test scores in aging. *Physical Therapy*.

[18] Woollacott MH, Woollacott MH, Shumway-Cook A (1989). Aging, posture control and movement preparation. *Development of Posture and Gait Across the Life Span*.

[19] Lord SR, Sherrington C, Menz H, Close JCT (2007). *Falls in Older People: Risk Factors and Strategies for Prevention*.

[20] Sheldon JH (1963). The effect of age on the control of sway. *Gerontologica Clinica*.

[21] Balogun JA, Akindele KA, Nihinlola J, Marzouk DK (1994). Age-related changes in balance performance. *Disability and Rehabilitation*.

[22] Balogun JA, Ajayi LO, Alawale F (1997). Determinants of single limb stance balance performance. *African Journal of Medicine and Medical Sciences*.

[23] Briggs RC, Gossman MR, Birch R, Drews JE, Shaddeau SA (1989). Balance performance among noninstitutionalized elderly women. *Physical Therapy*.

[24] Iverson BD, Gossman MR, Shaddeau SA, Turner ME (1990). Balance performance, force production, and activity levels in noninstitutionalized men 60 to 90 years of age. *Physical Therapy*.

[25] Choy NL, Brauer S, Nitz J (2003). Changes in postural stability in women aged 20 to 80 years. *Journals of Gerontology—Series A*.

[26] Du Pasquier RA, Blanc Y, Sinnreich M, Landis T, Burkhard P, Vingerhoets FJG (2003). The effect of aging on postural stability: a cross sectional and longitudinal study. *Neurophysiologie Clinique*.

[27] Prioli AC, Freitas Júnior PB, Barela JA (2005). Physical activity and postural control in the elderly: coupling between visual information and body sway. *Gerontology*.

[28] Dickin DC, Brown LA, Doan JB (2006). Age-dependent differences in the time course of postural control during sensory perturbations. *Aging Clinical and Experimental Research*.

